# Amputation neuroma derived from a remnant cystic duct 30 years after cholecystectomy: A case report

**DOI:** 10.1016/j.ijscr.2019.10.011

**Published:** 2019-10-12

**Authors:** Ryosuke Hirohata, Tomoyuki Abe, Hironobu Amano, Tsuyoshi Kobayashi, Akinori Shimizu, Keiji Hanada, Shuji Yonehara, Masahiro Nakahara, Hideki Ohdan, Toshio Noriyuki

**Affiliations:** a Department of Surgery, Onomichi General Hospital, Onomichi, Hiroshima, Japan; b Department of Gastroenterology, Onomichi General Hospital, Onomichi, Hiroshima, Japan; c Department of Pathology, Onomichi General Hospital, Onomichi, Hiroshima, Japan; d Department of Gastroenterological and Transplant Surgery, Applied Life Sciences, Institute of Biomedical and Health Sciences, Hiroshima University, Hiroshima, Japan

**Keywords:** AN, amputation neuroma, CT, computed tomography, MRCP, magnetic resonance cholangiopancreatography, EUS, endoscopic ultrasonography, FNA, fine needle aspiration, IDUS, intraductal ultrasonography, POCS, peroral cholangioscopy, BS, biliary stricture, OLT, orthotropic liver transplantation, LC, laparoscopic cholecystectomy, Case report, Amputation neuroma, Benign biliary disease, Remnant cystic ductal tumor

## Abstract

**Introduction:**

Amputation neuroma is difficult to diagnose preoperatively. Amputation neuroma arising from a remnant cystic duct after cholecystectomy is rare. Herein, we present a case of amputation neuroma derived from a remnant cystic duct along with a review of the literature.

**Presentaion of the case:**

A 60-year-old woman visited our hospital due to a tumor located in the hepatoduodenal ligament. A gallbladder adenoma was resected by open cholecystectomy 30 years prior. Endoscopic ultrasonography demonstrated branched intraductal papillary mucinous neoplasm of the pancreas and a tumor with a low-echoic pattern in the extrahepatic biliary system. Enhanced computed tomography revealed a 6-mm tumor in the artery phase. Surrounding lymph nodes were not swollen. Magnetic resonance cholangiopancreatography showed that the tumor presented with slightly high intensity on T2 weighted imaging. Operative findings revealed that the whitish nodule was moderately attached to surrounding tissues. The remnant cystic duct and the tumor could not be separated; however, no direct invasion toward common bile duct was observed. Rapid intraoperative pathological examination demonstrated that the tumor was a neuroma. The peration time was 251 min and blood loss was 80 ml. The patient was discharged nine days after surgery with no postoperative complications.

**Conclusion:**

It is difficult to distinguish amputation neuroma from malignant tumors because radiological findings of a neuroma mimic findings of malignancy. Intraoperative diagnosis is necessary to select an appropriate surgical procedure due to the difficulty of preoperative diagnosis.

## Highlights

• Amputation neuroma (AN) arising from a remnant cystic duct after cholecystectomy is rare.
• It is difficult to distinguish AN and malignant tumor because radiological findings of ANs mimic findings of malignancy.
• Intraoperative diagnosis is necessary to select an appropriate surgical procedure.

## 1 Introduction

Amputation neuroma (AN) is a reactive hyperplasia of nerve tissue that results from incomplete healing following trauma or surgery to a nerve. ANs are characterized by irregular growth of regenerated nerve bundle and fibrosis. ANs are non-neoplastic disorganized growths [1]. ANs form during the process of nerve healing. The abundant nerve supply around the biliary duct and ANs after cholecystectomy and liver transplantation has been reported. The incidence of ANs varies from 3% to 30% [2]. ANs are benign tumors, but radiological findings resemble those of cholangiocarcinomas, neuroendocrine tumors, and lymph node metastasis. Herein, we present a case of AN following surgical resection 30 years after cholecystectomy. The following case was in line with the SCARE criteria [3].

## 2 Case presentation

**Fig. 1:**
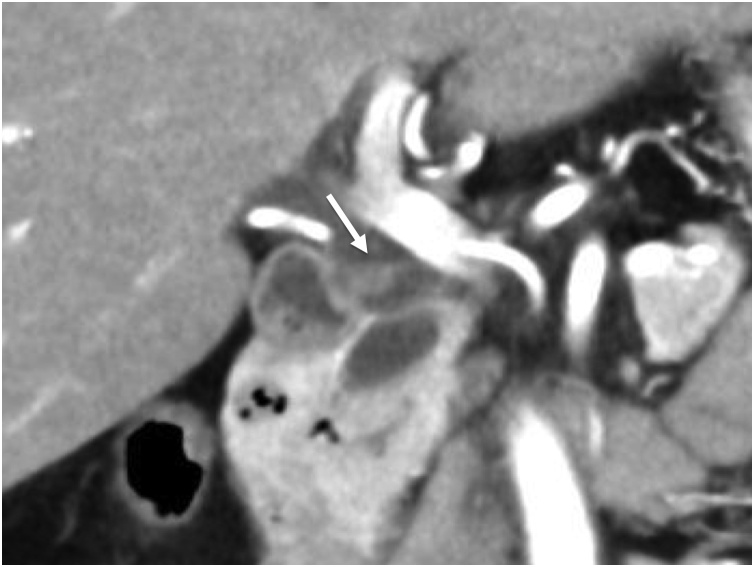
Enhanced computed tomography findings. Enhanced abdominal computed tomography showed the tumor (white arrow) adjacent to the common bile duct.

**Fig. 2:**
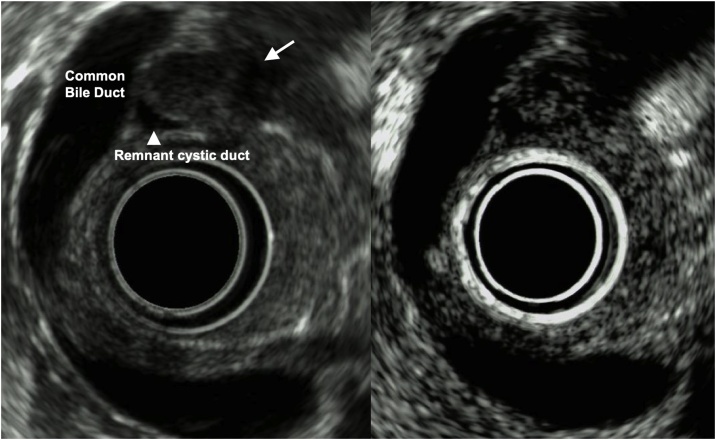
Endoscopic ultrasonography findings. Endoscopic ultrasonography demonstrated the tumor (white arrow) at the junction of the cystic duct. On Sonazoid-enhanced echo, the tumor was universally enhanced.

**Fig. 3:**
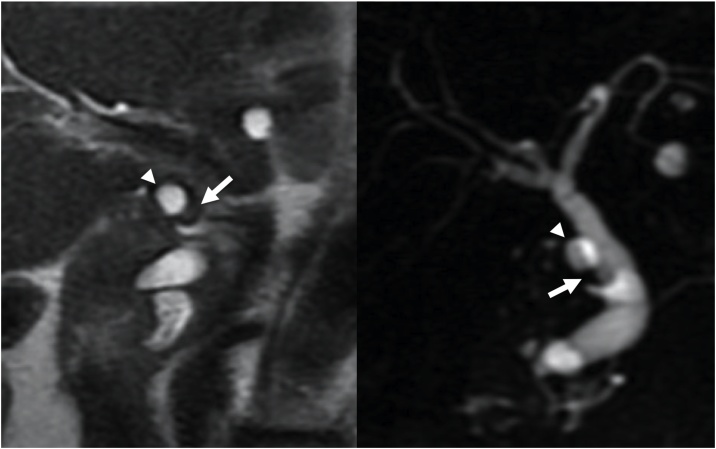
Magnetic resonance cholangiopancreatography findings. Magnetic resonance cholangiopancreatography findings show that the tumor (white arrow) had a slightly high signal on T2 weighted imaging. The remnant cystic duct was dilated by the tumor, which displayed high intensity on T2 weighted imaging (arrowhead).

**Fig. 4:**
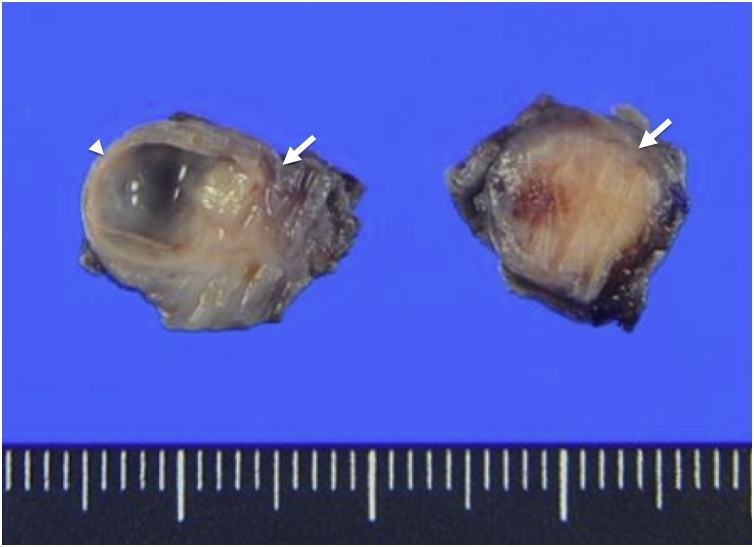
Macroscopic findings. Macroscopic findings had two components; the dilated remnant cyst with white bile (arrowhead), and the whitish main tumor with substantial neurofibrotic changes (white arrow).

**Fig. 5:**
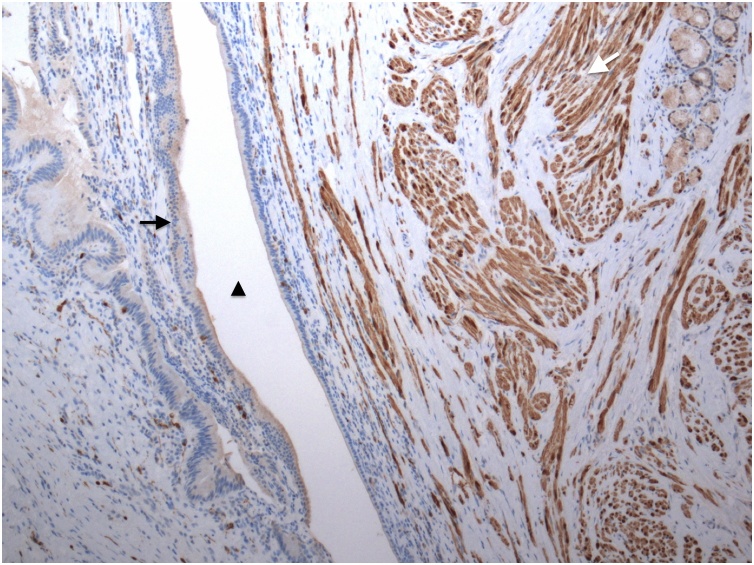
Pathological findings. The tumor was stained by anti S-100 antibody (white arrow). The wall of the cystic duct (black arrow) was compressed by the tumor, narrowing the intraductal space (black arrowhead).

A 60-year-old woman visited our hospital for evaluation of a tumor arising in a remnant cystic duct 30 years after cholecystectomy for gallbladder adenoma. Laboratory data, including tumor markers such as carcinoembryonic antigen and carbohydrate antigen 19-9, were within normal ranges. The patient had no chief complaint. Previous medical history included breast cancer that was completely resected three years prior to her visit. Since that time she had taken an aromatase inhibitor. Annual follow-up for breast cancer by contrasted computed tomography (CT) showed an intraductal papillary mucinous neoplasm (IPMN) in the pancreas head and an enhanced tumor image around the hepatoduodenal ligament (Fig. 1). Endoscopic ultrasonography (EUS) demonstrated branched IPMN of the pancreas and a residual cystic duct tumor. The tumor was located at the junction of the cystic duct and was enhanced with Sonazoid (Fig. 2). Endoscopic retrograde cholangiopancreatography indicated that the tumor had not invaded the common bile duct. Enhanced CT in the artery phase revealed a 6 mm round tumor. Surrounding lymph nodes were not swollen. Magnetic resonance cholangiopancreatography (MRCP) showed that the tumor presented with a slightly high signal on T2 weighted imaging, and the periphery remnant cystic duct of the tumor presented as a high-intensity lesion on T2 weighted imaging (Fig. 3). During surgery the tumor was located at the cutoff position of the remnant cystic duct and presented as a white nodule that adhered tightly to surrounding tissue. There was severe adhesion around remnant cystic duct and the hepatoduodenal ligament due to previous surgery. The remnant cystic duct and the tumor could not be separated; however, no invasion toward common bile duct was observed. Rapid intraoperative pathological examination demonstrated that the tumor was a neuroma. The operation time was 251 min, and blood loss was 80 ml. Macroscopic findings had two components; the dilated remnant cyst with white bile, and the whitish main tumor with substantial neurofibrotic changes (Fig. 4). Immunohistological examination revealed that the AN was compressing the cystic duct from the outside (Fig. 5). The patient was discharged nine days after surgery without any postoperative complications.

## 3 Discussion

ANs are traumatic neuromas that result from incomplete healing following trauma to the nervous system and often develop in limbs and neck [4,5]. ANs that form in the biliary system are relatively rare and are of two types. Symptomatic ANs in the biliary tract are typically accompanied by jaundice due to obstruction, along with pain from sympathetic nerves stimulation. Other ANs are non-symptomatic, as in the present case, and are mainly of concern due to difficulty in distinguishing them from malignancies.

As a result of widespread use of laparoscopic cholecystectomy (LC), reports of AN after LC have been increasing [6,7]. The period from initial cholecystectomy to the onset of AN varies from 2 to 40 years [8]. Our patient presented with an AN arising from a remnant cystic duct that mimicked radiological features of cholangiocarcinoma. In general, differential diagnoses of biliary tract tumors include cholangiocarcinomas, metastatic lymph nodes, neuroendocrine tumors, and inflammatory pseudotumors. Some cases of benign biliary tumors showed the same radiological features as malign tumors [9,10]. The proportion of benign biliary tract tumors is reported to be about 6% of all biliary tumors [11]. Therefore, distinguishing between benign and malignant biliary tract tumors is of great importance clinically.

Radiological features of ANs in magnetic resonance imaging studies reveal tumors with intermediate signal intensity on T1WI and high intensity on T2WI [12]. Increased fluorodeoxyglucose uptake of AN is reported [13], which makes precise preoperative diagnosis difficult. Enhanced CT findings are not effective for diagnosis since benign tumors show enhancement. Recently, the accuracy of endoscopic ultrasonography fine needle aspiration (EUS-FNA) is reported to be 68%–91%, especially in biliary stenosis [14]. However, in our case the tumor was located at the hepatic hilum; hence, EUS-FNA could cause severe complications such as biliary injury and massive bleeding. Shimura et al. reported the utility of intraductal ultrasonography (IDUS) for precise diagnosis of this disease [15]. In addition, Furukawa et al. reported the usefulness of peroral cholangioscopy (POCS) in ANs [16]. In our case, potential malignancy could not be discounted by IDUS and POCS was not compatible with the patient’s condition. Furthermore, ANs develop in the submucosal layer, and further study would be required to validate EUS-FNA and POCS for AN diagnosis.

Surgical resection would be the recommended approach for diagnosis and cure. In a typical case of AN, it is relatively easy to discount a malignant tumor without immunostaining [17], so avoiding excessive surgery using intraoperative rapid pathological diagnosis is important.

The biliary tract is richly innervated by the hepatic plexus, and ANs may occur after any operation that causes inflammation to hilar lesion. At this time, biliary stricture (BS) by AN after orthotropic liver transplantation (OLT) is the postoperative complication of greatest concern. Technical issues and ischemic-type lesions are considered as the leading causes of BS after OLT. AN is another cause of BS after OLT. The incidence of ANs after liver transplantation may be 0.5% to 30% [18,19]. Widespread use of LC for benign biliary disease will lead to finding patients with ANs derived from remnant cystic ducts. Special consideration for ANs is needed for patients with a previous medical history of LC.

## 4 Conclusion

It is difficult to distinguish ANs from malignant tumors using various radiological methods. POCS and IDUS could be helpful in making a precise diagnosis. A surgical approach is acceptable in the case of biliary ANs for both diagnosis and cure, considering the potential for biliary malignancy.

## Declaration of Competing Interest

None of the authors have any commercial or financial involvement in connection with this study that represents or appears to represent any conflicts of interest

## Sources of funding

This research received no specific grant from any funding agency in the public, commercial, or not-for-profit sectors.

## Ethical approval

Onomichi general hospital ethics review committee, OJH-2019-03

## Consent

Written informed consent was obtained from the patient for publication of this case report and any accompanying images.

## Author contribution

All authors in this manuscript contributed to the interpretation of data, and drafting and writing of this manuscript. Ryosuke Hirohata is first author of this paper. Tomoyuki Abe is corresponding author of this paper. Hironobu Amano, Masahiro Nakahara, Toshio Noriyuki, and Hideki Ohdan conceived and designed the study and drafted the manuscript. Ryosuke Hirohata first diagnosed. Ryosuke Hirohata, Akinori Shimizu, Keiji Hanada, and Masahiro Nakahara were engaged in patient’s care in our hospital including surgery. Hideki Ohdan and Tsuyoshi Kobayashi contributed to study concept, and review of the final manuscript and submission of the paper. All the authors read and approved the final manuscript.

## Registration of research studies

The manuscript does not report the result of an experimental investigation or research on human subjects.

## Guarantor

Tomoyuki Abe.

## Provenance and peer review

Not commissioned, externally peer-reviewed.
